# Safety and efficacy of water jet technology for internal thoracic artery harvesting in coronary artery bypass grafting: Initial results

**DOI:** 10.1016/j.xjtc.2025.10.021

**Published:** 2025-11-07

**Authors:** Yoshinori Nakahara, Akira Marui, Kohei Sumi, Ryogen Yun, Makoto Ono, Tomohiro Iwakura, Tatsuya Murai

**Affiliations:** a Department of Cardiovascular Surgery, Sakakibara Heart Institute, Tokyo, Japan; b Department of Pathology, Sakakibara Heart Institute, Tokyo, Japan

**Keywords:** coronary artery bypass, internal thoracic artery, water jet, tissue dissection, graft harvesting

## Abstract

**Objective:**

To evaluate the safety and feasibility of water jet technology for internal thoracic artery (ITA) harvesting in coronary artery bypass grafting.

**Methods:**

We retrospectively reviewed 5 consecutive patients who underwent coronary artery bypass grafting with ITA harvesting using water jet technology. ITAs were harvested using water jet technology at 10 bar pressure and anastomosed to the target coronary arteries. Primary end points included technical success rate, intraoperative flow measurements, and postoperative graft patency by computed tomography. Secondary end points included histologic evaluation of excess graft segments when available.

**Results:**

Nine ITAs were successfully harvested in all 5 cases without complications. Mean harvest time was 29.7 ± 5.7 minutes, with excellent graft flow of 30.9 ± 13.1 mL/minutes. Postoperative computed tomography confirmed 100% graft patency (9/9 ITAs). Histologic examination of 6 ITAs with available excess segments revealed minimal tissue change with microhemorrhages and minimal perivascular coagulation, without thermal injury to medial or intimal layers.

**Conclusions:**

Water jet technology demonstrates feasibility and safety for ITA harvesting with excellent clinical outcomes and minimal tissue trauma. This thermal-free approach may offer advantages over conventional energy-based harvesting methods.

## Graphical Abstract

**Figure:**
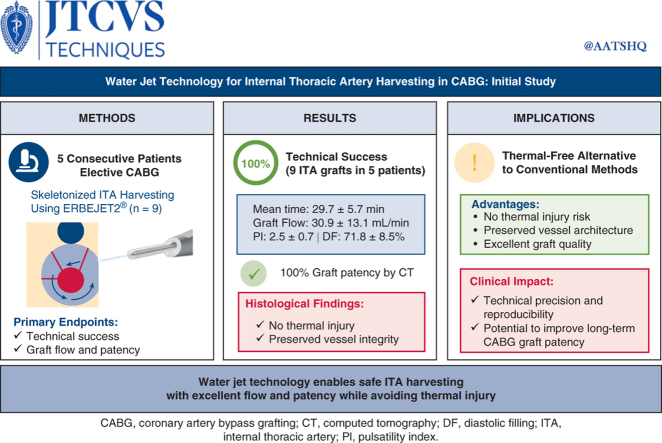

**Figure:**
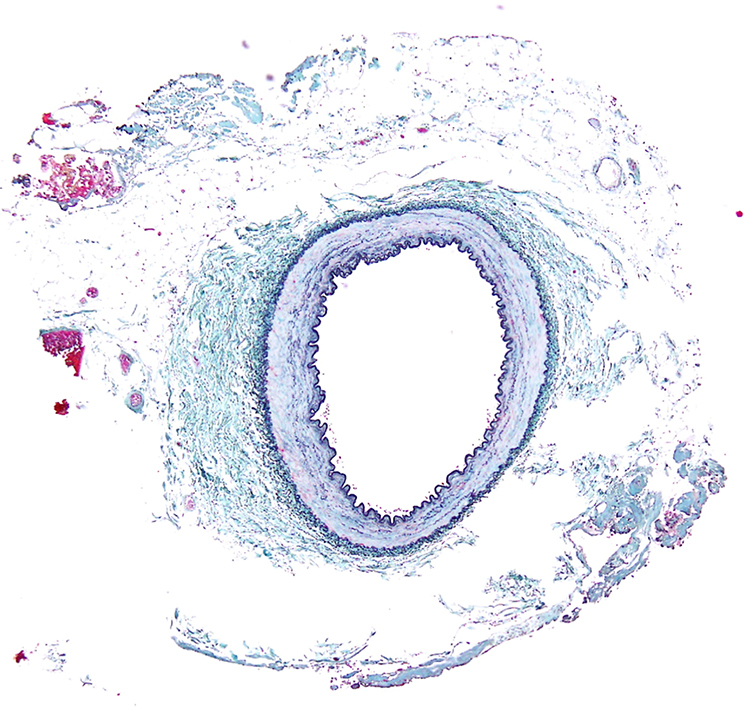
Water jet−harvested ITA showing fluid layer around ITA and minimal tissue change.

**Central Message**

Water jet technology enabled safe ITA harvesting in all 5 cases with excellent flow and 100% patency while avoiding thermal injury.

**Perspective**

Traditional ITA harvesting uses thermal energy devices that risk vessel damage. This pilot study demonstrates water jet technology as a promising thermal-free alternative, achieving 100% technical success with excellent graft outcomes and minimal histologic tissue change, potentially improving CABG results.

Coronary artery bypass grafting (CABG) remains the gold standard treatment for complex coronary artery disease, with the internal thoracic artery (ITA) being the conduit of choice because of its superior long-term patency.^1^ The quality of the harvested ITA directly impacts both early and late outcomes of CABG, making meticulous harvesting technique essential for preserving graft integrity.^2^

Current harvesting methods pose inherent limitations. Electrocautery and ultrasonic devices, although widely used,^1^ both generate thermal energy during tissue dissection. A recent meta-analysis showed a trade-off: ultrasonic scalpels reduce endothelial injury but require significantly longer harvest times compared with electrocautery.^3^ Most importantly, both methods inevitably produce thermal injury, risking damage to the vessel wall and surrounding tissues.^4^

Water jet (WJ) technology offers a fundamentally different approach by using pressurized saline to separate tissue planes without thermal energy, thereby preserving vital structures.^5^ A commercially available WJ device for surgical applications is the ERBEJET 2 system (ERBE). Our experimental histologic study using swine ITAs demonstrated zero thermal injuries across 19 histologic specimens,^6^ and successful applications have been reported in other surgical specialties such as nerve-sparing radical prostatectomy.^7^ However, no data have been available on WJ technology for ITA harvesting in human CABG so far.

Therefore, the present study provides the first clinical evaluation, to our knowledge, of WJ technology for ITA harvesting in CABG. We assessed technical success, graft flow characteristics, postoperative patency, and histologic findings to establish initial evidence for this thermal-free harvesting approach in cardiac surgery.

## Methods

### Study Design and Patient Selection

This study was conducted at Sakakibara Heart Institute between July and September 2024. The study protocol was approved by the institutional review board (approval number: 24-020, approval date: June 24, 2024), and written informed consent was obtained from all patients for publication of study data. Patients scheduled for CABG using an ITA were eligible for inclusion. All ITAs harvested using WJ technology were included in the analysis of technical success, intraoperative flow characteristics, and postoperative patency. Histologic evaluation was performed on excess graft segments when available after completion of anastomosis.

### Surgical Technique

ITA harvesting was performed using the WJ device (ERBEJET 2) with a pressure setting of 10 bar as previously described.^6^ The surgical technique is demonstrated in Video 1. The ITA was harvested in a skeletonized fashion from the subclavian vein to its bifurcation. WJ dissection created the initial tissue plane and protective fluid cushion around the ITA. After this initial WJ dissection, remaining fibrous or avascular tissue attachments were divided with microscissors under direct vision. All visible ITA side branches, regardless of size, were basically divided using surgical clips to completely avoid thermal injury to the ITA and its branches. Electrocautery was used exclusively for pleural incision and management of surrounding adipose tissue or small branches when sufficiently distant from the ITA main trunk to avoid thermal injury. This hybrid approach combines the thermal-free advantage of WJ dissection with the precision of selective sharp dissection for residual tissue.

### Outcome Measures

Primary end points included the following: (1) technical success of ITA harvesting; (2) intraoperative graft flow measurements; and (3) postoperative graft patency assessed by computed tomography (CT). Secondary end points included histologic findings of the harvested specimens, especially coagulation and bleeding and dissection.

### Histologic Assessment

The distal excess segments of the harvested ITAs were collected for histologic evaluation. The specimens were fixed in 10% formalin solution, and tissue blocks including the lateral branches were extracted from all ITAs. Cross-sections were prepared and stained with hematoxylin and eosin for general histologic examination. In addition, these specimens were processed with Elastica Masson staining. Microscopic photographs were taken of all sections and used to evaluate (1) coagulation and bleeding of the ITA, including the perivascular tissue; and (2) extent of tissue damage (eg, dissection).

All segments were ITAs less than 1 cm in length, and to eliminate the effects of focal dissection of the media during clipping or cutting and artifacts during specimen preparation, 4 or more sections were taken, and findings that were consistent across all sections were defined as positive findings. All histologic evaluations were performed by a blinded pathologist.

### Statistical Analysis

Data were analyzed using R software, version 4.4.3 (R Foundation for Statistical Computing; https://www.r-project.org/). Continuous variables are presented as mean ± standard deviation and categorical variables as frequencies and percentages.

## Results

### Patient Characteristics

Between July and September 2024, 5 consecutive patients (2 male patients, 69.0 ± 9.1 years old) underwent elective CABG with ITA harvesting using WJ technology. A total of 9 ITAs (5 left internal thoracic arteries [LITAs] and 4 right internal thoracic arteries [RITAs]) were harvested using WJ technology in these 5 cases and all were successfully used for grafting. All LITAs were used as in situ LITA-left anterior descending artery grafts, whereas RITAs were used as either free grafts or in situ grafts to target coronary arteries. Six ITAs had excess distal segments remaining after anastomosis that were available for histologic examination; the remaining 3 RITAs (cases 1, 3, and 4) were fully used for grafting with no surplus tissue for analysis. Patient characteristics and surgical details are shown in Table 1.

**Table 1:** Patient characteristics and surgical details for water jet-assisted ITA harvesting (n = 9 ITAs in 5 patients)

Case	Age, y	Sex	Primary diagnosis	Surgical procedure	ITA graft configuration
1	63	Male	MR, EAP	Mitral valve repair + CABG (4 grafts)	LITA → LAD, RITA → diagonal
2	79	Female	AS, ICM	Aortic valve replacement + CABG (3 grafts)	LITA → LAD
3	70	Female	OMI, EAP	OPCAB (3 grafts)	LITA → LAD, free RITA → LCx
4	76	Female	EAP	OPCAB (3 grafts)	LITA → LAD, RITA → diagonal
5	57	Male	OMI, ICM	OPCAB (6 grafts)	LITA → LAD, free RITA → proximal and distal LCx

*ITA*, Internal thoracic artery; *MR*, mitral regurgitation; *EAP*, effort angina pectoris; *CABG*, coronary artery bypass grafting; *LITA*, left internal thoracic artery; *LAD*, left anterior descending artery; *RITA*, right internal thoracic artery; *AS*, aortic stenosis; *ICM*, ischemic cardiomyopathy; *OMI*, old myocardial infarction; *OPCAB*, off-pump coronary artery bypass; *LCx*, left circumflex artery.

### Operative Outcomes

WJ-assisted ITA harvesting achieved a 100% success rate (n = 6) with all 9 ITAs successfully harvested without intraoperative complications. Mean harvest time was 29.7 ± 5.7 minutes (range, 21-39 minutes). Intraoperative transit time flow measurement revealed excellent graft quality across all 9 ITAs: mean flow 30.9 ± 13.1 mL/min, pulsatility index 2.5 ± 0.7, and diastolic filling 71.8 ± 8.5%. Detailed intraoperative data for each ITA are shown in Table 2.

**Table 2:** Intraoperative graft performance and postoperative patency of water jet−harvested ITAs (n = 9)

Case	Harvested ITA	Harvest time, min	Mean graft flow, mL/min	Pulsatility index	Diastolic filling, %	Postoperative CT angiography	Histologic analysis
1	LITA	35	33	3.3	81	Patent	Available
1	RITA	21	36	3.3	75	Patent	Not available∗
2	LITA	28	52	1.9	77	Patent	Available
3	LITA	35	12	3.4	74	Patent	Available
3	RITA	24	15	1.8	69	Patent	Not available∗
4	LITA	30	26	1.9	62	Patent	Available
4	RITA	39	24	2.2	64	Patent	Not available∗
5	LITA	27	35	2.4	84	Patent	Available
5	RITA	28	45	2.0	60	Patent	Available
Mean ± SD		29.7 ± 5.7	30.9 ± 13.1	2.5 ± 0.7	71.8 ± 8.5	100% patency	6/9 (67%)

All 9 ITAs were successfully harvested using water jet technology and demonstrated satisfactory hemodynamic parameters with 100% patency rate on postoperative CT angiography. Histologic analysis was performed on 6 ITAs that had excess segments available after completion of anastomosis. *ITA*, Internal thoracic artery; *CT*, computed tomography; *LITA*, left internal thoracic artery; *RITA*, right internal thoracic artery.

∗ RITAs in cases 1, 3, and 4 were fully used for grafting with no excess segments available for histologic examination.

### Postoperative Course

All patients recovered without complications and were discharged from the hospital. Postoperative CT angiography confirmed 100% graft patency for all 9 ITAs (Table 2).

### Histologic Findings

**Figure 1:**
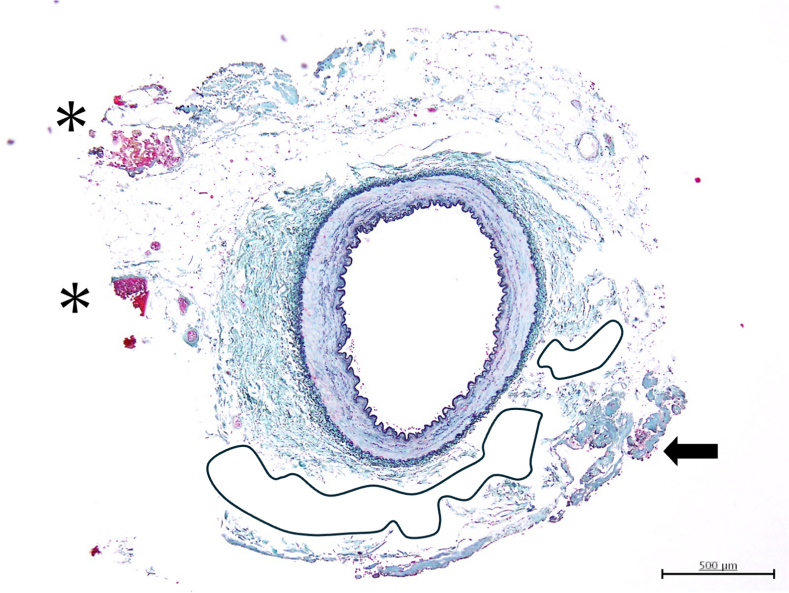
Representative histologic examination of internal thoracic artery harvested using water jet technology (case 5 left internal thoracic artery). Cross-sectional view demonstrates minimal perivascular tissue change with preserved vessel architecture. *Asterisk* (∗) indicates areas of minor hemorrhage in the adventitial layer. *Black arrows* indicate coagulation areas from limited electrocautery use. The *outlined areas* show perivascular fluid accumulation created by water jet dissection. Note the intact intimal and medial layers with no thermal injury. Similar findings were observed in all 6 examined internal thoracic artery specimens. Elastica Masson staining, × 100 magnification.

Histologic evaluation was performed on 6 ITAs (5 LITAs and 1 RITA from case 5) that had excess segments available after anastomosis. The remaining 3 RITAs (cases 1, 3, and 4) were fully used for grafting without surplus tissue for analysis. WJ technology preserved arterial wall integrity without intimal-to-medial injury or dissection in all examined specimens. Representative specimens (Figure 1) show minimal perivascular tissue change with preserved vessel architecture. WJ dissection caused perivascular fluid accumulation as the primary tissue response. Secondary findings included minimal coagulation in 83% (5/6) of specimens and microhemorrhage in 100% (6/6), both limited to the outer adventitial layer without vessel wall compromise (Table 3).

**Table 3:** Histologic findings in water jet−harvested ITAs (n = 6 of 9 total ITAs)

Case	Harvested ITA	Adventitial coagulation∗	Adventitial hemorrhage†	Intimal/medial injury
1	LITA	Present	Present	None
2	LITA	Present	Present	None
3	LITA	Present	Present	None
4	LITA	Present	Present	None
5	LITA	Present	Present	None
5	RITA	Absent	Present	None
Summary		5/6 (83%)	6/6 (100%)	0/6 (0%)

Histologic examination was performed on 6 ITAs (all 5 LITAs and 1 RITA from case 5) that had excess segments available after anastomosis. The remaining 3 RITAs (cases 1, 3, and 4) were fully used for grafting without surplus tissue for analysis. All examined specimens preserved vessel wall integrity with no thermal injury to intimal or medial layers. *ITA*, Internal thoracic artery; *LITA*, left internal thoracic artery; *RITA*, right internal thoracic artery.

∗ Minor coagulation limited to outer adventitial layer, attributed to minimal electrocautery use.

† Minor hemorrhage confined to perivascular tissue without vessel wall compromise.

## Discussion

**Figure 2:**
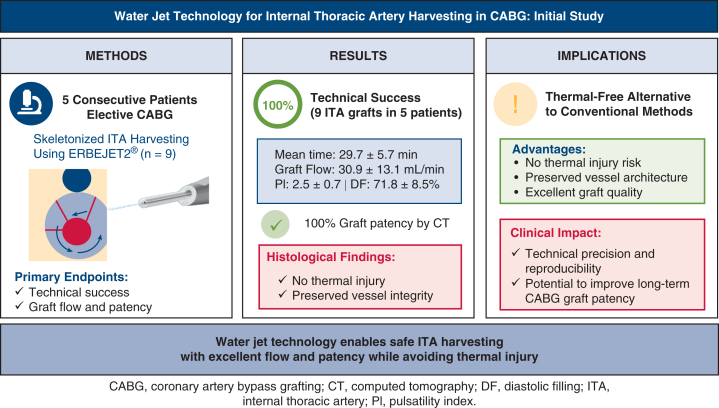
Water jet technology for ITA harvesting. This study evaluated water jet−assisted ITA harvesting in 5 consecutive patients who underwent CABG, with 9 ITAs harvested. Technical success was achieved in 100% of cases (9/9 ITAs) with excellent graft flow (30.9 ± 13.1 mL/min) and 100% postoperative patency. Histologic examination of 6 available ITA segments revealed no thermal injury with preserved vessel wall integrity. Water jet technology offers a thermal-free alternative to conventional harvesting methods with potential advantages for long-term graft patency. *ITA*, Internal thoracic artery; *CABG*, coronary artery bypass grafting.

This initial study successfully demonstrated the clinical feasibility and safety of WJ technology for ITA harvesting in CABG (Figure 2). We achieved 100% technical success with all 9 ITAs successfully harvested and used for grafting in 5 consecutive cases with excellent graft flow (30.9 ± 13.1 mL/min) and 100% confirmed patency on postoperative CT angiography. Histologic analysis of 6 available ITA specimens revealed preservation of vessel wall integrity with only minor perivascular changes—microhemorrhages from WJ dissection and minimal coagulation from limited electrocautery use—without any thermal damage to the intimal or medial layers. Of particular importance was the formation of a protective fluid cushion around the ITA, as demonstrated in Figure 1. The pressurized saline creates a fluid layer that physically separates the ITA from surrounding tissues and surgical instruments, acting as a mechanical buffer against direct contact trauma and compression injury to the endothelium during dissection. This protective effect may extend beyond thermal injury prevention to reduce mechanical endothelial damage that can occur even with careful instrument manipulation. However, this proposed mechanism requires validation through controlled studies with endothelial function assessment. Although this study presents only short-term perioperative outcomes, the preservation of vessel wall integrity may potentially contribute to improved long-term graft patency.^8^

The successful translation from experimental to clinical application demonstrates the technology's practical viability. Our mean harvest time of 29.7 ± 5.7 minutes compares favorably with reported ultrasonic harvesting times while maintaining superior hemodynamic parameters.^3^ The manageable learning curve suggests that WJ technology can be readily adopted in clinical practice, aligning with previous experimental research showing its ability to achieve precise tissue dissection.^5^

WJ technology's primary advantage lies in its thermal-free approach. Unlike electrocautery and ultrasonic devices that inevitably produce heat damage,^1^ our histologic analysis found no thermal injury in any specimen. The observed microhemorrhages remained confined to perivascular tissues, corroborating our previous experimental data showing significantly lower coagulation rates (26.3% vs 96.0%, *P* < .01) compared to electrocautery.^6^ Schurr and colleagues^9^ similarly demonstrated that WJ dissection causes the least tissue trauma among energy-based surgical tools. Although no clinical adverse events occurred in this series, continued monitoring remains essential.

Despite these advantages in tissue preservation, WJ technology has inherent limitations that must be considered. WJ dissection created the initial tissue plane, but sharp dissection with microscissors remained necessary for dividing residual fibrous attachments. The technology's selective preservation of small vessels (0.1-0.3 mm at 20 bar pressure),^6^ while protecting delicate structures, necessitates additional hemostatic measures. In our experience, surgical clips were required for branch management, and limited electrocautery was used for surrounding tissues and small branches distant from the ITA. This hybrid approach, though requiring additional instrumentation, ultimately ensures minimal heat exposure to the graft itself while maintaining the thermal-free advantage of WJ dissection. We acknowledge that WJ technology does not address hemoclip-related complications such as clip dislodgement or improper application, which remain important technical considerations in ITA harvesting. Future technical refinements should focus on optimizing branch management while maintaining the technology's atraumatic benefits.

WJ technology represents a shift from thermal to mechanical tissue dissection in cardiac surgery. We acknowledge that meticulous conventional technique using sharp dissection, judicious cautery placement, and systematic clipping can achieve excellent results in skilled hands. However, WJ technology may offer advantages in technical reproducibility and reduced mechanical trauma. Raja and Dreyfus^10^ emphasized that harvesting technique directly influences long-term graft performance, making vessel preservation during harvesting critically important. A post hoc analysis of the Arterial Revascularization Trial found that the technically more demanding skeletonized ITA harvesting was associated with a higher rate of major adverse cardiac events at 10 years compared to the pedicled technique.^11^ This difference was most pronounced for surgeons with less experience, highlighting that technical precision and reproducibility have significant impact on long-term outcomes. The protective fluid cushion created by WJ dissection may reduce mechanical endothelial trauma and potentially improve consistency across varying surgeon experience levels, though direct comparative studies are essential to validate these hypothesized advantages. The technology's successful application in nerve-sparing prostatectomy^7^ demonstrates its potential for preserving delicate structures across surgical specialties. For CABG procedures where graft quality determines long-term outcomes, this thermal-free approach may offer a new option for atraumatic vessel harvesting.

Several limitations require acknowledgment while interpreting these promising results. The small sample size (5 patients, 9 ITAs) limits statistical power and generalizability. Most importantly, we lack direct histologic comparison with conventional harvesting techniques in the same patient population, although our previous experimental study demonstrated significantly lower thermal injury with WJ technology.^6^ Histologic analysis was only possible in 6 of 9 ITAs due to complete use of 3 RITAs for grafting without excess segments. We also lack long-term follow-up data to assess the impact of preserved vessel integrity on graft patency beyond the perioperative period. Additionally, the learning curve and cost-effectiveness remain unquantified, and the workflow efficiency of incorporating WJ technology into routine practice needs validation across multiple centers.

WJ technology proved both feasible and safe for clinical ITA harvesting. All 9 ITAs achieved technical success with excellent hemodynamic parameters and 100% postoperative patency. Histologic examination of available specimens demonstrated preserved vessel architecture without thermal injury. Although larger comparative studies with long-term follow-up are essential, these results establish WJ technology as a promising thermal-free alternative that may enhance graft quality in CABG. Future applications may include adaptation of WJ technology for minimally invasive ITA harvesting, as the protective fluid cushion could potentially facilitate visualization and reduce trauma in confined thoracoscopic or robotic surgical spaces.

## Conflict of Interest Statement

The authors reported no conflicts of interest.

The *Journal* policy requires editors and reviewers to disclose conflicts of interest and to decline handling or reviewing manuscripts for which they may have a conflict of interest. The editors and reviewers of this article have no conflicts of interest.

## Supplemenatry Data

Video 1 Water jet−assisted internal thoracic artery harvesting. Skeletonized ITA harvesting using water jet technology (ERBEJET 2, 10 bar pressure). The technique demonstrates thermal-free dissection with preservation of vessel integrity, followed by clip division of side branches and minimal electrocautery use limited to tissues distant from the main ITA trunk. Video available at: https://www.jtcvs.org/article/S2666-2507(25)00481-X/fulltext.
