# Supplementary figures and images for: Safety and efficacy of water jet technology for internal thoracic artery harvesting in coronary artery bypass grafting: Initial results
Source: JTCVS Tech. 2025 Nov 7;35:102146. doi: 10.1016/j.xjtc.2025.10.021 (PMC12881812; doi:10.1016/j.xjtc.2025.10.021)

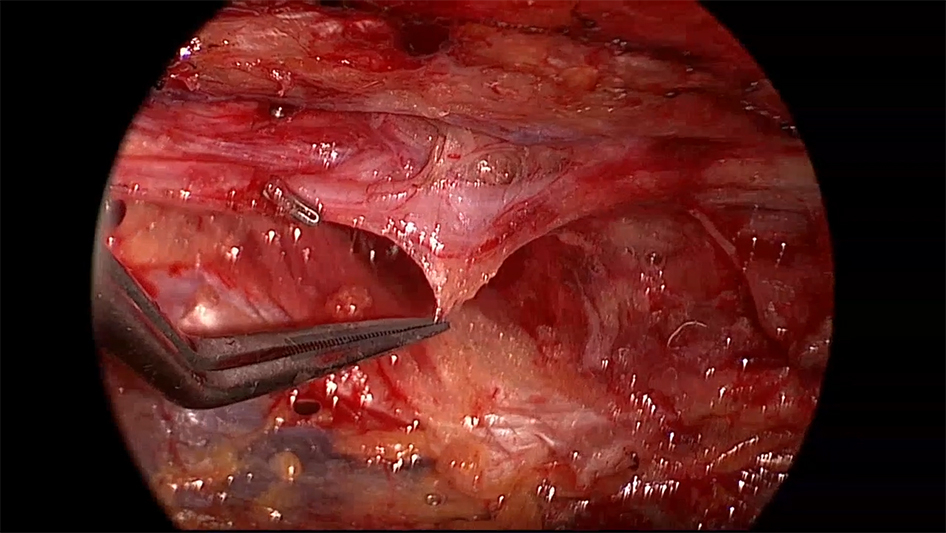

Supplement: Video 1 — Water jet−assisted internal thoracic artery harvesting. Skeletonized ITA harvesting using water jet technology (ERBEJET 2, 10 bar pressure). The technique demonstrates thermal-free dissection with preservation of vessel integrity, followed by clip division of side branches and minimal electrocautery use limited to tissues distant from the main ITA trunk. Video available at: https://www.jtcvs.org/article/S2666-2507(25)00481-X/fulltext. [file fx2.jpg]
